# Physiochemical characteristics of date pollen extract-hybrid yoghurt as a functional dairy food modulates hypothyroidic symptoms in modeled rats

**DOI:** 10.1038/s41598-025-29389-1

**Published:** 2025-12-06

**Authors:** Laila K. Hassan, Marwa H. El-Azma, Mostafa Elaaser, Doaa G. EL-Sahra, Ghada M. Elqattan, Mostafa S. A. Khattab, Mahmoud Abd El-Aziz, Khaled G. Abdel-Wahhab

**Affiliations:** 1 Dairy Department, Institute of Food Industries and Nutrition Research, National Research Centre, Giza, 12622 Egypt; 2 Medical Physiology Department, National Research Centre, Giza, 12622 Egypt; 3 Modern University for Technology and Information, Cairo, Egypt

**Keywords:** Date pollen ethanolic extract, Hybrid yoghurt, Physiochemical characteristics, Hypothyroidism, Antioxidant activity, Anti-inflammatory, Biochemistry, Biological techniques, Biotechnology, Chemical biology

## Abstract

Yoghurt is a widely consumed dairy product across the globe. It possesses healing properties and qualities that are significant for human health. This study aimed to evaluate the properties of date pollen ethanolic extract-hybrid yoghurt (DPEE-HY) on hypothyroidism as a functional dairy food. Standardized cow’s milk was hybridized with 1 or 2% of DPEE, while the control yoghurt (CY) was not hybridized. Adult male Wistar albino rats were used to evaluate the pharmaceutical impacts of DPEE-HY on propylthiouracil (PTU)-induced hypothyroidic rats. The rats were randomly assigned to four groups as follows: (1) healthy animals ingested 1 ml of liquefied non-hybrid yoghurt, acting as control; (2) healthy animals ingested with DPEE-HY (250 mg/kg/day) for 30 days; (3) induced hypothyroidic rats; (4) hypothyroidic rats administered with DPEE-HY for 30 days. The results revealed that DPEE-HY (1 and 2%) showed pH values, acetaldehyde and diacetyl concentrations, and WSN/TN ratios similar to those of control yoghurt; however, it exhibited strong antioxidant activity that was proportional to the DPEE concentration, as well as the highest hardness, and slightly lower viscosity. Administration of hypothyroidic rats with DPEE-HY markedly improved the pathophysiological status of the thyroid gland, as it significantly upregulated levels of T3 and T4, alongside a downregulation in TSH level. The oxidative status was improved by DPEE-HY which markedly decreased the levels of oxidative indicators (MDA and NO) and increased the values of antioxidant markers (GSH, SOD, GPx, and CAT). DPEE-HY also exhibited immunomodulatory effects by reducing the levels of anti-inflammatory markers (TNFα and IL-1β) while simultaneously increasing the INFγ level. Additionally, it significantly improved hepato-renal function and lipid profile, as well as the histological architecture of the thyroid in comparison to the corresponding findings of hypothyroid rats. In conclusion, DPEE-HY showed acceptable physicochemical characteristics, and performed thyroid-modulatory efficiency that could be mediated via the antioxidant properties that antagonized oxidative stress and apoptosis of thyroid tissue; the enhancement effect of DPEE-HY on hypothalamic-pituitary-thyroid axis preserved thyroid-genesis that in turn maintained normal thyroid functions; consequently, hybridization of yoghurt with DPEE could be a potent modulatory dairy product for management of hypothyroid disorder.

## Introduction

Milk and dairy products are a major source of multiple nutrients that meet the dietary requirements for protein, calcium, magnesium, phosphorus, potassium, zinc, selenium, vitamin A, riboflavin, vitamin B12, and pantothenic acid. However, in many countries, dairy consumption is declining and moving away from the recommended amount and the potential health advantages of milk and dairy products have been questioned^1^. Dairy products are particularly advantageous for fostering health in countries where regular consumption of milk, cheeses, and fermented milk beverages constitutes a substantial portion of the traditional diet^2^. Dairy products are popular because of their high nutritional value, appealing sensory appeal, and health-promoting properties^3^. Although some research casts questions on the health advantages of dairy products, many studies show that consuming dairy products has positive health effects. According to a recent large global cohort study from 21 countries across 5 continents, dairy consumption is related to a lower risk of mortality and severe CVD events^4^. Furthermore, the overall consumption of dairy products, particularly yoghurt, was inversely linked to the incidence of type 2 diabetes, according to the meta-analysis done by Gijsbers et al^5^.. There is strong evidence that the risk of hypertension is inversely correlated with the overall amount of dairy products consumed and that the risk of T2D and hypertension is correlated with the consumption of low-fat dairy products and yoghurt. In addition, findings indicate that the consumption of various dairy products is either neutrally or favorably correlated with clinical aspects of cardiovascular health^6^.

Oxidative stress results in reactive oxygen species, which are waste products of aerobic metabolism^7^. Hypothyroidism results in a decrease in thyroid hormones. The condition might lead to symptoms like exhaustion, cold intolerance, weight gain, and persistent constipation. Untreated hypothyroidism can lead to metabolic problems like diabetes mellitus and cardiovascular disorders^8^. Numerous studies have demonstrated that hypothyroidism is associated with a hypometabolic state, which may lead to a decrease in oxidative damage and the generation of free radicals^9^. Reactive oxygen species are extremely harmful and can lead to a number of diseases. Thus, antioxidants need to speed up their removal through a number of cellular processes^10^. The bioactivity of dairy products for health has been well investigated; in particular, numerous research studies have found that cheese possesses antioxidant properties due to the presence of phenolic compounds in dairy products^11^. Several studies have shown that the complicated interaction between phenolic compounds and milk proteins is responsible for the antioxidant effects; however, due to the low levels of polyphenolic compounds in dairy products, this antioxidant effect may be minimal. The addition of natural substances, such as herbs, to a range of dairy products has been well researched as a way to improve bioactivity for human health^12^.

The pollen of date palm (*Phoenix dactylifera* L.) is a fine powder material produced by male flowering date palms, and it is a good source of minerals, enzymes, hormones, fatty acids, protein, amino acids, dietary fiber, and vitamins A, B, C, D, and E^13,14^ as well as phenolic acid, carotenoids, flavonoids, and polyphenols, along with oestrone, oestradiol, and oestriol^15^. Due to its high concentration of bioactive compounds, it is thought to be an effective natural and functional food supplement. These compounds play important roles as strong antioxidants, antimicrobials, anti-inflammatories, anti-toxins, and hepatoprotective agents, as well as health-promoting nutrients that are used all over the world as dietary supplements^16,17^. The investigation of hybrid yoghurts that combine dairy and plant-based derivatives has generally been spurred by a perceived shift in consumer preferences towards plant-based diets, which is fuelled by factors like lactose intolerance, allergies, and vegetarian trends; however, dairy products are still easily accessible^18,19^. This trend is evident worldwide, with plant-based yoghurt substitutes being viewed as reasonably priced dairy alternatives, especially in developing countries^20^. The present study constitutes the attempt to prepare acceptable physicochemical characteristics of date pollen ethanolic extract-hybrid-yoghurt (DPEE-HY), as well as to investigate its modulatory potency on modeled hypothyroidic-rats in a trial to determine its efficiency to mitigate the symptoms of hypothyroidism.

## Materials and methods

### Materials

Cows’ milk was obtained from the farm of the National Research Centre, Giza, Egypt. Dried date pollen (*Phoenix dactylifera* L.) was obtained from a local market; then it was identified and authenticated by a professional botanist and found carrying a taxonomy ID of 42,345. The pollen was fine-powdered and kept in a refrigerator at 4 °C until use. The USA-made skim milk powder (SMP, 34.23% protein) was purchased from a local market in Cairo, Egypt. Stock cultures were obtained from the Dairy Microbiology Lab. (National Research Centre, Egypt) supplied the starter cultures for *Lactobacillus delbrueckii* ssp. *bulgaricus* and *Streptococcus thermophiles*. To separately activate each strain, three transfers were made into modified MRS, followed by three additional transfers into sterile 11% reconstituted SMP. Semicarbazide hydrochloride, 2,2’-azino-bis (3-ethylbenzothiazoline-6-sulphonic acid) (ABTS), and 2,2-diphenyl-1-(2,4,6-trinitrophenyl)-hydrazinyl (DPPH) were purchased from Sigma-Aldrich (St. Louis, MO, USA). Analytical-grade chemicals and reagents were obtained from different sources.

## Methods

### Date pollen ethanolic extract (DPEE)

The fine-powdered date pollens were soaked in a suitable volume of 70% ethanol (1:7) at room temperature for two days with stirring. The mixture was centrifuged at 3000 xg for 10 min, filtered, and the residue was then re-extracted with the same amount of solvent. Two ethanol filtrates were combined and evaporated using a rotary vacuum evaporator (Rotary Evaporator BüchiR-210, Flawil, Switzerland) under reduced pressure at 50 °C to complete removal of solvent. The freeze dryer was used for lyophilization of the aqueous residues; then the dry date pollen ethanolic extract (DPEE) was kept at −20 °C until used.

### Phenolic compounds

The HPLC analysis of the phenolic compounds in DPEE was conducted using an Agilent 1260 series, USA. Phenolic compounds were separated using the Eclipse C_18_ column (4.6 mm x 250 mm ID x 5 μm). The mobile phase consisted of water (A) and 0.05% trifluoroacetic acid in acetonitrile (B) at a flow rate of 0.9 ml/min. The mobile phase was programmed consecutively in a linear gradient as follows: 0 min (82% A), 0–5 min (80% A), 5–8 min (80–60% A), 8–12 min (60% A), 12–15 min (60–85% A) and 15–16 min (82% A). An auto-injector (10 µL) was used to inject the phenolic standard solution and DPEE into the apparatus. At 280 nm, the multi-wavelength detector was observed. Phenolic compounds were detected using UV absorption at 280 nm. The retention time of each compound and a comparison with standards under the same conditions were used for components identifications.

### Yoghurt making

Fresh cows’ milk was standardized to 15% total solids (TS) using skim milk powder and divided into three equal portions. One portion contained no DPEE as a control yoghurt (CY), while the other portions were hybridized with DPEE [human equivalent dose (HED) was after conversion of the used animal dose using animal-HED conversion equation] at rates of either 1 or 2% (w/w) to produce DPEE-HY_1_ (1%) that contains HED, and DPEE-HY_2_ (2%), respectively. Each batch was preheated to 65 °C, homogenized for 5 min at 21,000 rpm using a laboratory homogenizer (Polytron^®^ PT 10–35 GT, Kinematica, Switzerland), heated for 5 min to 90 °C, and then cooled to 42 °C. A 2.0% starter culture consisting of *L. delbrueckii* subsp. *bulgaricus* and *S. thermophilus*, in a 1:1 ratio, was added and thoroughly mixed before the mixture was poured into 120 mL food-grade plastic cups. The cups were then incubated at 42 °C until the pH reached 4.6–4.7. On days 1, 7, 14, and 21 of the storage period, the yoghurt samples were examined after being quickly cooled and kept at 5 ± 1 °C.

### Chemical analysis

The pH of the yoghurt samples was measured during the different storage periods using a glass electrode laboratory pH meter (HANNA, equipment, Portugal). The Conway microdiffusion-semicarbazide method^21^ was used to determine the concentrations of acetaldehyde and diacetyl, as flavor compounds, in the yoghurt samples. The degree of proteolysis that took place in yoghurt samples during storage was assessed using the water-soluble nitrogen/total nitrogen ratio (WSN/TN ratio). The concentration of water-soluble nitrogen in the yoghurt samples was determined using methods described by^22^.

### Antioxidant activity

According to Brand-Williams et al.^23^ and Re et al.^24^, stable DPPH radicals (DPPH) and stable ABTS radicals (ABTS) tests were used to measure the antioxidant activity of the yoghurt samples in their whey. A 3.9 mL of DPPH or ABTS working (OD = 0.7) solution was mixed with 100 µL of the yoghurt-filtered whey. After a half-hour incubation period at room temperature in the dark, the degree of decolorization was measured using a spectrophotometer. The DPPH radical-scavenging assay was conducted at 517 nm, while the ABTS radical-scavenging assay was conducted at 734 nm. Similarly, DPPH and ABTS without whey were made as control solutions. The scavenging activities of DPPH and ABTS radicals were calculated using the following formula.

Antioxidant activity (%) = [(A_0_ – A_1_)/A_0_] x 100.

A_0_ is the absorbance of the control (DPPH or ABTS solution), and A_1_ is the absorbance of the sample.

### Apparent viscosity

The apparent viscosity of the yoghurt samples was measured at 12 rpm using a Brookfield digital viscometer (Model DV-II, Canada) fitted with measuring spindle 04, as explained by Shazly et al.^25^. Before being placed in a 100 ml glass cylinder for measurement, the yoghurt sample was gently spun five times in a clockwise direction using a plastic spoon. Following a 30-second spin at 7 ± 1 °C, the apparent viscosity was then measured; the torque was > 10% and < 90%. The apparent viscosity values were expressed in Pascal-second (Pa.s).

### Biological study

#### Experimental design

Adult male Wistar albino rats (150–180 g) were obtained from the Animal Colony, National Research Centre, Giza, Egypt. Before starting the experiment, the animals were housed in suitable plastic cages for one week to be acclimatized. Excess tap water and standard rodent pellets were always available; all animals received human care in compliance with the standard institutional criteria for the care and use of experimental animals, however the study proposal was approved by the ethical committee of Faculty of Science, Al-Azhar University (Assiut Branch) with a number (AZHAR 15/2024, May 2024), however all methods are reported in accordance with ARRIVE guidelines.

#### Induction of hypothyroidism

Suitable number (25) of rats was subjected to induction of hypothyroidism which was done by drinking the animals with 0.05% propylthiouracil in water (w/v) for 6 weeks as described by Sahoo et al.^26^; however, blood samples were withdrawn from the animals and tested for T4, T3, and TSH to ensure the occurrence of hypothyroidism. After occurrence of the hypothyroidism, the induced-hypothyroidic rats besides healthy ones were randomly arranged into four groups (10 rats each) as follows: (1) healthy animals administered with 1 ml of liquefied control yoghurt (CY) and acting as normal control; (2) healthy animals ingested daily with 1 ml of liquefied DPEE-HY  (equivalent to 250 mg DPEE/kg) for 30 days^27^; (3) untreated hypothyroidic animals; (4) hypothyroidic animals treated with 1 ml of liquefied DPEE-HY for 30 days.

#### Sampling of blood and tissue

All animals were fasted at the end of the experiment, and blood samples were withdrawn post-anesthesia (through isoflurane inhalation), allowed to clot, and cool-centrifuged; then, the sera were separated, divided into aliquots, and kept at −80 °C until biochemical analyses could be carried out. Soon after the blood withdrawal, the animals were sacrificed by sudden decapitation, and each thyroid was removed and immersed in 10% v/v formalin-saline buffer for histological examination.

#### Biochemical and immunological measurements

Thyroid serum hormonal profile (total T4, total T3, and TSH levels) and immune-inflammatory and regulatory markers (TNF-α, IL-1β, IL-6, INF-γ, CAM, and TGF-β) were assessed using rats’ reagent ELISA kits (Sunlong Biotech Co., Hang Zhou, China). Serum urea, creatinine, ASAT, ALAT, total cholesterol, triglycerides, NO, MDA, GSH, GPx, CAT, and SOD values were measured spectrophotometrically using reagent kits of Biodiagnostic, Giza, Egypt.

#### Histopathology

After blood collection, the thyroid gland was of each animal dissected out (with a part of the trachea) and immediately kept in 10% buffered formalin–saline solution for a later histopathological examination. The histological preparations were performed in accordance to Bancroft & Layton^28^, as thyroid was Sects. (3–4 mm thick) were sliced, dehydrated in ethanol at several concentrations, cleaned in xylene, and stained with hematoxylin and eosin stain to examine the overall structure of the tissue under light microscope. Also, the sections were meticulously examined and scored based on criteria outlined in the studies by Akkurt et al.^29^ and Arslan et al.^30^ . Each section was evaluated for the presence and severity of follicular degeneration (FD), decrease in colloidal fluid (DCF), fibrosis, atypical follicle epithelium (AFE), and mononuclear cell infiltration (MCI). The scoring system employed ranged from 0 to 3, where score 0 indicated no structural damage, score 1 indicated minor damage, score 2 indicated moderate damage, and score 3 indicated severe damage. This uniform application of the scoring system ensured consistency and accuracy in the assessment of the histopathological changes.

### Statistical analysis

Statistical analysis was conducted using SAS (2008)^31^ software and the GLM technique. Both one- and two-way analyses of variance (ANOVA) were performed, along with Duncan’s multiple comparison method, to compare the means. A probability threshold of *p* < 0.05 was established for determining statistical significance.

## Results and discussion

### Phenolic compounds in date pollen ethanolic extract

Table 1 presents the concentrations of phenolic compounds identified in the date pollen ethanolic extract (DPEE), as determined by HPLC analysis. Methyl gallate emerged as the most abundant phenolic compound in DPEE, with a concentration of 3357.98 µg/g, accounting for 63.68%. It was followed by naringenin, which had a concentration of 711.92 µg/g, representing 13.50%. The gallic, chlorogenic, syringic, and rosmarinic acids were also identified in considerable concentrations in DPEE, with 311.04, 259.51, 193.66, and 100.24 µg/g, respectively. Low levels of ellagic acid, ferulic acid, coumaric acid, quercetin, and rutin were found, but vanillin and daidzein were found in trace concentrations. Hesperetin, catechins, kaempferol, caffeic, and cinnamic acids, on the other hand, were not detected. These results contradicted those of Shahin et al.^32^ , who found that the date pollen (variety El-Hayani) extracted with 70% ethanol had the highest contents of rutin (15.43%), apigenin (12.26%), and coumarin (11.12%), followed by chlorogenic acid, o-coumaric acid, gallic acids, quercetin, and luteolin. Another study conducted in Tunisia showed that the most abundant compounds in the DPEE of Kerkennah were vanillic acid (56.23%), gallic acid (23.17%), and caffeic acid (9.64%). However, coumarin (55.76%) and epicatechin (29.25%) were the most abundant compounds in date pollen of Tozeur, but caffeic acid was not detected^33^. This suggests that the date palm variety, extraction method, and solvent type all have an impact on the types and concentrations of phenolic compounds found in the DPEE.

**Table 1:** HPLC analysis of the phenolic compounds in date pollen ethanolic extract.

Phenolic compounds	Concentrations (µg/g)	(%)
Gallic acid	311.04	5.90
Chlorogenic acid	259.51	4.92
Catechin	ND	-
Methyl gallate	3357.98	63.68
Coffeic acid	ND	-
Syringic acid	193.66	3.67
Rutin	57.52	1.09
Ellagic acid	81.25	1.54
Coumaric acid	25.47	0.48
Vanillin	11.99	0.23
Ferulic acid	78.23	1.48
Naringenin	711.92	13.50
Rosmarinic acid	100.24	1.90
Daidzein	4.31	0.08
Querectin	80.48	1.53
Cinnamic acid	ND	-
Kaempferol	ND	-
Hesperetin	ND	-

ND, not detected.

### DPEE-hybrid yoghurt

#### Biochemical changes

The biochemical changes in DPEE-hybrid yoghurt (DPEE-HY) during 21 days of storage at 5 ± 1 °C are displayed in Table 2. The DPEE-HY and the control yoghurt (CY) did not differ significantly (*p* > 0.05) in pH values or acetaldehyde and diacetyl concentrations. Although the WSN/TN ratio was numerically lower in the yoghurt hybrid with 2% DPEE (DPEE-HY_2_) than in the yoghurt hybrid with 1% DPEE (DPEE-HY_1_) and CY, the differences were not significant (*P* > 0.05). During storage, the acetaldehyde concentration and pH value both considerably dropped; however, the WSN/TN ratio and diacetyl concentration increased (*P* < 0.05). The pH decreased as a result of bacterial metabolic activity, which may break down lactose and produce more organic acids, such as lactic and acetic acids^34^. The acetaldehyde concentration decreased during storage due to the metabolic pathway, which produces diacetyl from it^35^. According to^36^, alcohol dehydrogenase activity also converts acetaldehyde into ethanol, which causes the component to decrease. Stojanovska et al.^37^ found that on the first day, fermentation produced the highest concentrations of acetaldehyde in all the dairy samples. Additionally, whether or not probiotic bacteria (*B. bifidum* and/or *L. rhamnosus*) are present, brown yoghurt exhibited a significant reduction in acetaldehyde concentrations during storage^38^. During the yoghurt fermentation process, the starter cultures also produce various protein enzymes that break down milk proteins into peptides, which serve as a nitrogen source for the bacteria. However, in both CY and DPEE-HY1, the WSN/TN ratio exhibited a significant increase by day 7, whereas in DPEE-HY2, the increase was notable on day 15^39^. Overall, the DPEE-HY had no discernible effect on the proteolysis or flavor compounds of the final product when compared to the CY.

**Table 2:** Biochemical changes in the DPEE-hybrid yoghurt during 21 days of storage at 5 ± 1 °C in comparison to the control yoghurt.

Items	Storage period (day)	Yoghurt treatments		
		CY	DPEE-HY_1_	DPEE-HY_2_
pH	1	4.71^Aa^ ± 0.06	4.65^Aa^ ± 0.05	4.62^Aa^ ± 0.05
	7	4.50^Ab^ ± 0.12	4.48^Ab^ ± 0.03	4.49^Aa^ ± 0.07
	15	4.25^Ac^ ± 0.04	4.22^Ac^ ± 0.07	4.21^Ab^ ± 0.03
	21	4.17^Ac^ ± 0.09	4.19^Ac^ ± 0.10	4.18^Ab^ ± 0.04
Acetaldehyde (µmol/100 g)	1	78.07^ABa^ ± 4.77	84.23^Aa^ ± 2.10	72.39^ABa^ ± 1.92
	7	57.69^Ab^ ± 2.39	52.92^Bb^ ± 1.35	55.94^Ab^ ± 0.99
	15	44.45^Bc^ ± 3.46	38.79^Bc^ ± 2.74	42.25^Ab^ ± 2.27
	21	41.18^Ac^ ± 1.51	37.68^Ac^ ± 1.53	37.65^Ad^ ± 3.15
Diacetyl (µmol/100 g)	1	17.75^Ac^ ± 0.92	21.75^Ab^ ± 0.25	17.92^Ab^ ± 0.34
	7	21.75^Ac^ ± 1.52	22.67^Ab^ ± 4.0	21.75^Ab^ ± 1.17
	15	34.83^Bb^ ± 2.35	43.25^Aa^ ± 2.42	37.25^Ba^ ± 1.25
	21	45.00^Aa^ ± 4.56	45.08^Aa^ ± 1.75	44.67^Aa^ ± 2.00
WSN/TN ratio (%)	1	8.04^Ab^ ± 0.26	7.87^Ab^ ± 0.26	7.87^Ab^ ± 0.21
	7	9.21^Aa^ ± 0.36	9.45^Aa^ ± 0.25	8.68^Aab^ ± 0.18
	15	9.93^Aa^ ± 0.29	9.84^Aa^ ± 0.35	9.07^Aa^ ± 0.51
	21	9.97^Aa^ ± 0.41	9.68^Aa^ ± 0.75	9.17^Aa^ ± 0.60

The data are shown as mean ± SE; Means with similar lowercase letters in the same column and similar uppercase letters in the same row do not differ significantly at P < 0.05; CY, control yoghurt; DPEE-HY1, hybrid yoghurt with 1% date pollen ethanolic extract; DPEE-HY2, hybrid yoghurt with 2% date pollen ethanolic extract.

#### Antioxidant activity

**Fig. 1:**
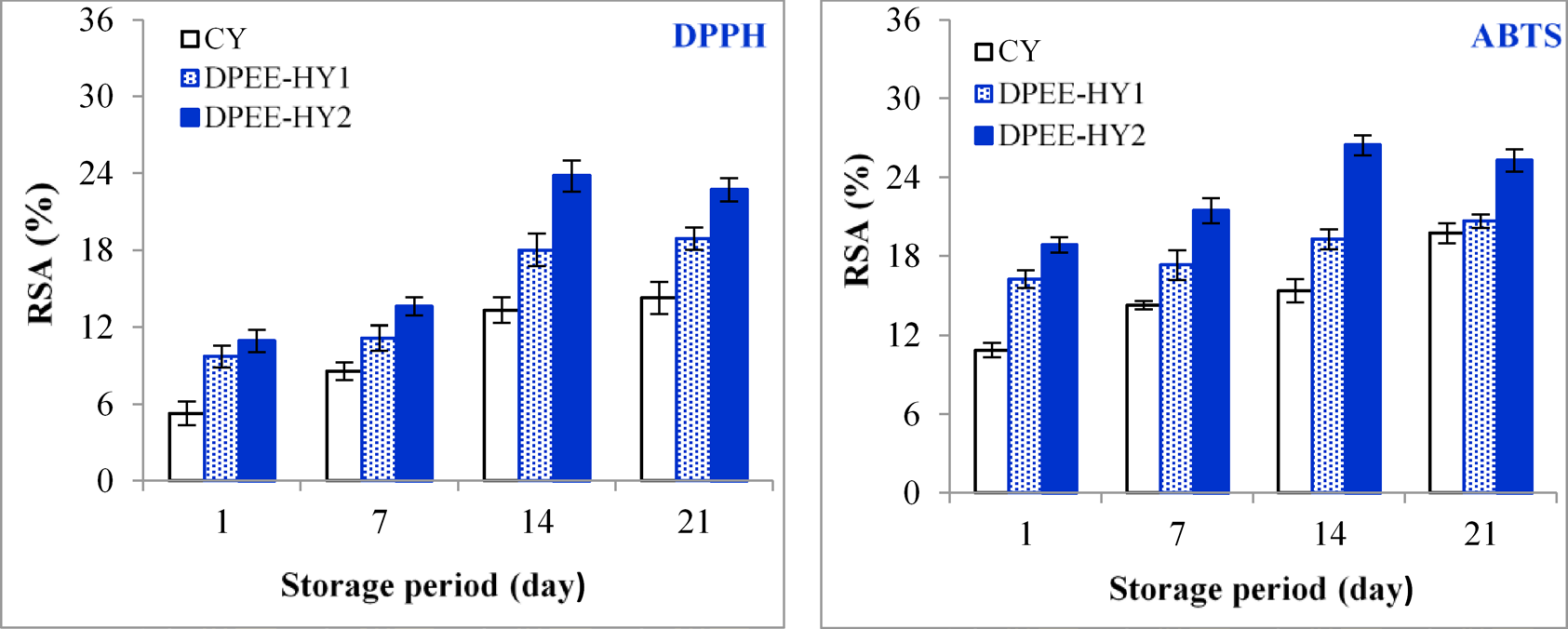
Antioxidant activity in the DPEE-hybrid yoghurt during 21 days of storage at 5 ± 1 °C in comparison to the control yoghurt.

As illustrated in Fig. 1, DPEE-HY exhibited higher antioxidant activities against DPPH and ABTS radicals compared to CY. The elevated antioxidant activity may be attributed to an increase in antioxidant components, including phenols, unsaturated fatty acids, bioactive proteins, and vitamins in the DPEE. Polyphenols possess potent antioxidant properties that can reduce oxygen free radicals and alleviate oxidative stress in the human body^40^. El-Kholy et al.^41^ found that Egyptian date palm pollen is abundant in unsaturated fatty acids (ω−3, ω−6, and ω−9) and phenolic compounds, particularly quercetin, gallic acid, and rutin. These compounds are enhanced by ethanol extraction and demonstrate high antioxidant activity (IC_50_ 11.76 mg/g). Ryu et al.^42^ discovered that methyl gallate, which is the main phenolic part of DPEE, had strong antioxidant and lipid peroxidation-inhibitory properties, making it a valuable natural source of antioxidants. Moreover, the increase in antioxidant activity was proportionate to the DPEE concentration and time of storage. Antioxidant activity against DPPH and ABTS radicals increased significantly after one week in CY and after two weeks in DPEE-HY_1_ and DPEE-HY_2_ (*P* < 0.05). Additionally, the changes in antioxidant activity over storage were similar to those found in brown yoghurt prepared from buffalo’s milk^38^ and probiotic yoghurt made from ewe’s milk^25^. During storage, the antioxidant activity of yoghurt may increase as lactic acid bacteria continuously break down milk proteins into smaller bioactive peptides. These peptides are effective in reducing oxidative damage by scavenging free radicals and binding to metal ions^43,44^. Other possible explanations for this increase include the production of organic acids, which leads to an increase in acidity. This process promotes the degradation of complex phenolic compounds into smaller, more antioxidant molecules, as well as potential interactions between milk proteins and other compounds^43,44^.

Protein hydrolysis may be related to an increase in antioxidant activity during storage. According to^45^, the degree of hydrolysis improves casein’s strong antioxidant activity. Starter cultures are capable of producing low molecular weight bioactive peptides, most of which come from β-casein and less from αs1 casein^46^.

#### Physical properties

**Fig. 2:**
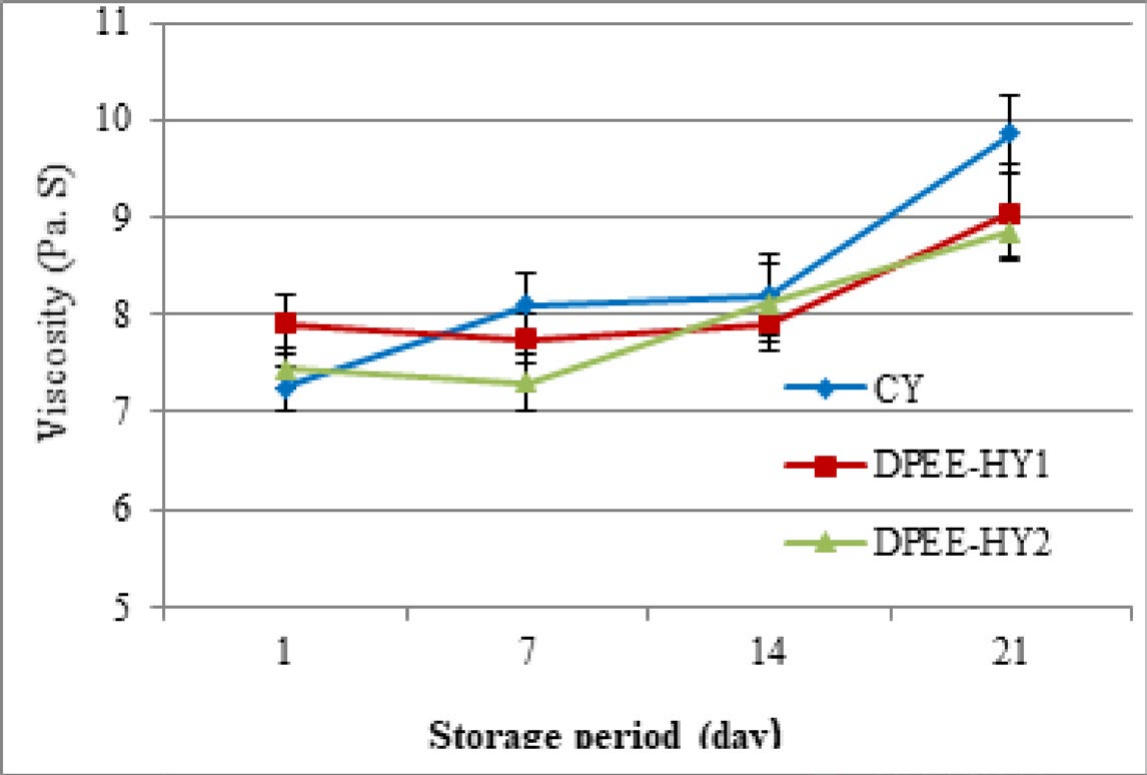
Apparent viscosity of the DPEE-hybrid yoghurt during 21 days of storage at 5 ± 1 °C in comparison to the control yoghurt.

The physical properties of DPEE-HY, specifically apparent viscosity and texture profile attributes, were evaluated in comparison to CY. On day 1, DPEE-HY_1_, which was hybridized with 1% DPEE, exhibited the highest apparent viscosity compared to DPEE-HY_2_ and CY, as illustrated in Fig. 2. However, on day 7 and subsequently, CY displayed the highest viscosity. This suggests that the viscosity of yoghurt may have been negatively impacted by the addition of DPEE, particularly at higher concentrations. The viscosity of yoghurt is shown to decrease when polyphenolic preparations are added, as demonstrated by^47^. Milk proteins and polyphenol complexes have the potential to induce protein unfolding and the formation of insoluble complexes, which may be responsible for this effect^48,49^. Izadi et al.^50^ indicated that the gel structure of yoghurt could weaken due to the addition of phytosterol emulsion. In addition, a slight, gradual increase in apparent viscosity was found throughout storage until day 15, but it became more noticeable on day 21 (*p* < 0.05). According to Shaan et al.^51^, protein rearrangements and protein-protein interactions can lead to an increase in apparent viscosity over time. As the pH decreases, there is reduced electrostatic repulsion, which enhances casein-casein attraction due to increased hydrophobic interactions. These components contribute to greater gel stiffness by promoting the formation and strength of binding, thereby elevating storage modulus values^52^.

As opposed to viscosity, yoghurt became harder when DPEE was added (*p* < 0.05); however, the increase in hardness was not statistically significant (*p* > 0.05) as the concentrations of the additive increased (Table 3). However, the DPEE-HY_1_ exhibited the lowest levels of cohesiveness, springiness, and gumminess until day 15 of storage, after which an increase was observed. The cohesiveness and gumminess of DPEE-HY_2_, on the other hand, increased at a high concentration on day 15 and then started to decrease. El-Kholy et al.^41^ reported that yoghurt fortification with date-pollen extract enhanced hardness, cohesiveness, and gumminess but had a negative impact on springiness. Throughout the storage period, both hardness and springiness progressively increased. Hardness showed a significant increase on day 21, while springiness showed a significant increase on days 15 and 21 (*p* < 0.05). A reduction in pH during storage may have caused the gel to contract, resulting in increased firmness and contributing to the rise in springiness and hardness^53^.

**Table 3:** Texture profile attributes of the DPEE-hybrid yoghurt during 21 days of storage at 5 ± 1 °C in comparison to the control yoghurt.

Items	Storage period (day)	Yoghurt treatments		
		CY	DPEE-HY_1_	DPEE-HY_2_
Hardness (N)	1	1.83^Ab^ ± 0.08	2.11^Ab^ ± 0.13	2.21^Ab^ ± 0.07
	7	1.89^Ab^ ± 0.07	2.20^Ab^ ± 0.15	2.30^Ab^ ± 0.13
	15	2.31^Ab^ ± 0.15	2.51^Ab^ ± 0.11	2.62^Ab^ ± 0.12
	21	2.72^Aa^ ± 0.16	3.02^Aa^ ± 0.10	3.01^Aa^ ± 0.14
Cohesiveness (B/A area)	1	0.39^Aa^ ± 0.03	0.33^Aa^ ± 0.01	0.45^Aa^ ± 0.02
	7	0.47^Aa^ ± 0.02	0.33^Aa^ ± 0.03	0.47^Aa^ ± 0.03
	15	0.39^Aa^ ± 0.02	0.31^Aa^ ± 0.03	0.39^Aa^ ± 0.02
	21	0.46^Aa^ ± 0.01	0.50^Aa^ ± 0.02	0.40^Aa^ ± 0.01
Springiness (mm)	1	0.50^Ac^ ± 0.03	0.42^Ac^ ± 0.01	0.54^Ac^ ± 0.08
	7	0.61^Ac^ ± 0.02	0.53^Abc^ ± 0.04	0.67^Ab^ ± 0.03
	15	0.77^Ab^ ± 0.06	0.68^Ab^ ± 0.04	0.79^Ab^ ± 0.02
	21	1.23^Aa^ ± 0.11	1.64^Aa^ ± 0.03	1.21^Aa^ ± 0.07
Gumminess (N)	1	0.71^Aa^ ± 0.02	0.79^Aa^ ± 0.04	0.88^Aa^ ± 0.06
	7	0.84^Aa^ ± 0.04	0.73^Aa^ ± 0.06	0.87^Aa^ ± 0.07
	15	0.74^Aa^ ± 0.08	0.70^Aa^ ± 0.07	0.72^Aa^ ± 0.08
	21	0.83^Aa^ ± 0.07	1.03^Aa^ ± 0.11	0.71^Aa^ ± 0.05

The data are shown as mean ± SE; Means with similar lowercase letters in the same column and similar uppercase letters in the same row do not differ significantly at p < 0.05

### Biological study

#### Thyroid profile

Table 4 of the study shows the ability of DPEE-HY to modulate the hypothyroid hormonal profile. The results declared that hypothyroidic rats exhibited a significant decrease in serum levels of both T3 and T4 coupled with a marked elevation in TSH level. This finding runs in line with previous ones^54,55^. Interestingly, treatment of hypothyroidic-modeled rats with DPEE-HY markedly elevated T3 and T4 while efficiently reducing TSH levels. This effect could be mechanized through the prevention of propylthiouracil binding with thyroid peroxidase, which consequently facilitates the conversion of iodide to iodine, then enhances iodine incorporation onto the phenol rings of thyroglobulin’s tyrosine, and spontaneous degradation of thyroglobulin to thyroxine (T4) and triiodothyronine (T3). It was stated that phytosterol-containing treatments induced the increased activity of thyroid glands, as evidenced by elevated levels of its hormones^56,57^; however,  it was reported that date pollen is composed of high phytosterols, flavonoids, and saponins.

**Table 4:** Serum values of thyroid profile of healthy, hyperthyroidic, and DPEE-hybrid yoghurt treated animals.

	Control	DPEE-HY	Hypothyroidic	Hypothyroidic ~ DPEE-HY
T3 (ng/ml)	0.812 ± 0.083	0.867 ± 0.031	0.391 ± 0.021^*^	0.719 ± 0.039^#^
T4 (ug/dl)	56.6 ± 2.320	60.12 ± 2.460	26.04 ± 0.677^*^	45.31 ± 1.177^#^
TSH (uIU/mL)	0.623 ± 0.037	0.666 ± 0.022	0.968 ± 0.061^*^	0.772 ± 0.035^#^

The data are shown as mean ± SE; the symbol * indicates a significant difference from the control group, while the symbol # indicates a significant difference from the hypothyroidic group at p< 0.05

#### Oxidative stress status

The oxidative stress marker values of the experimental groups are shown in Table 5. Results showed a significant rise in oxidative markers (MDA and NO) and a noticeable decline in antioxidant markers (GSH, SOD, GPx, and CAT) for the hypothyroidic group as compared with the control group. Generally, DPEE-HY markedly improved the antioxidants’ capacity; however, treatment of hypothyroidic rats with DPEE-HY resulted in a remarkable decrease in the oxidative markers (MDA & NO) associated with an obvious increase in the antioxidant parameters (GSH, SOD, GPx, and CAT) compared to the untreated hypothyroidic group. Hypothyroidism is associated with an imbalance in oxidative stress markers due to reduced thyroid hormone levels, that resulted in decreased metabolic activity and accumulation of reactive oxygen species (ROS)^58^. The oxidative markers (MDA and NO) indicate lipid peroxidation and nitrosative stress, respectively^59^. The body’s defense against oxidative stress involves both enzymatic and non-enzymatic antioxidants. Key enzymatic antioxidants (SOD, GPx, and CAT) work to neutralize ROS and prevent cellular damage; however, GSH plays a crucial role in maintaining redox balance^58^. DPEE-HY significantly reduced the levels of oxidative markers (MDA and NO) and markedly increased the antioxidant battery (GSH, SOD, GPx, and CAT) in hypothyroidic-DPEE-HY treated rats compared to untreated hypothyroidic rats. This suggests that DPEE-HY has potent antioxidant properties that can mitigate oxidative damage and reflects improved antioxidant defense mechanism, which helps in neutralizing ROS and protecting cellular integrity^60^. The antioxidant effects of DPEE-HY can be attributed to its rich content of bioactive compounds, such as flavonoids, phenolics, and vitamins, that are known to enhance the body’s antioxidant capacity through their ability to scavenge free radicals and upregulate the expression of antioxidant factors, thereby reducing oxidative stress^61^.

**Table 5:** Hepatic values of oxidative stress markers of healthy, hyperthyroidic, and DPEE-hybrid yoghurt treated animals.

	Control	DPEE-HY	Hypothyroidic	Hypothyroidic ~ DPEE-HY
MDA (µmol/g)	2.17 ± 0.04	2.11 ± 0.03	6.24 ± 0.09^*^	3.64 ± 0.05^#^
NO (µmol/g)	188 ± 6.75	179 ± 4.11	394 ± 7.22^*^	269 ± 4.67^#^
GSH (nmol/g)	9.31 ± 0.093	7.66 ± 0.072	2.67 ± 0.034^*^	6.17 ± 0.07^#^
SOD (U/g)	2895 ± 57	2904 ± 61	937 ± 38^*^	2010 ± 58^#^
GPx (U/g)	12.35 ± 0.19	12.94 ± 0.18	5.22 ± 0.11^*^	9.87 ± 0.27^#^
CAT (U/g)	4.21 ± 0.68	4.67 ± 0.81	1.37 ± 0.26^*^	3.91 ± 0.68^#^

The data are shown as mean ± SE; the symbol * indicates a significant difference from the control group, while the symbol # indicates a significant difference from the hypothyroidic group at p < 0.05

#### Immune inflammatory and regulatory markers

Table 6 shows the results of immune inflammatory and regulatory markers of the studied experimental groups; the results showed that the hypothyroidic group recorded a significant rise in serum TNF-α, IL-1β, IL-6, CAM, and TGF-β levels associated with a remarkable drop in INF-γ level as compared with the healthy control group. These findings are concomitant with those reported by previous studies^62–64^. It was stated that IFN-γ may be a humoral mediator in the pathogenesis of hypothyroidism *in vivo*. Moreover, IFN-γ seems play a crucial role in tissue repair; therefore, it regulates many organ functions in mammals through participation^65^. In contrast, administration of hypothyroidic rats with DPEE-HY resulted in a significant modulation in the mentioned immune markers compared to the corresponding values of the untreated hypothyroidic group. The introduction of DPEE appears to modulate the immune response effectively by improving immune markers via reducing the elevated inflammatory markers; DPEE-HY seems to restore immune balance closer to the levels of healthy controls. The active components of DPEE may promote a more balanced Th1/Th2 response, therefore inhibit the production of pro-inflammatory cytokines and adhesion molecules and might enhance INF-γ levels^66,67^^68^. Also, these active constituents could target specific signaling pathways, likely inhibiting the production of pro-inflammatory cytokines (TNF-α, IL-1β, IL-6), reducing systemic inflammation possibly via direct modulation of NF-κB signaling, a key regulator of inflammatory responses^69^. In addition, many immune disorders, including hypothyroidism, are linked to oxidative stress; however, DPEE-HY herein may function as an antioxidant, neutralizing free radicals and preventing inflammatory damage; therefore, DPEE-HY may rebalance immune responses, preventing chronic inflammation^70^.

**Table 6:** Serum values of immune inflammatory and regulatory markers of healthy, hyperthyroidic, and DPEE-HY treated animals.

	Control	DPEE-HY	Hypothyroidic	Hypothyroidic ~ DPEE-HY
TNF-α (pg/ml)	109.4 ± 3.93	104.6 ± 1.93	191.9 ± 4.39^*^	129.6 ± 2.48^#^
IL-1β (pg/ml)	38.73 ± 1.85	34.91 ± 1.15	62.25 ± 2.42^*^	45.03 ± 1.74^#^
IL-6 (pg/ml)	36.73 ± 1.98	35.74 ± 1.85	61.09 ± 3.67^*^	44.96 ± 2.44^#^
INF-γ (pg/ml)	605.7 ± 0.52	614.3 ± 6.44	426.8 ± 5.73^*^	568.1 ± 7.12^#^
CAM (ng/ml)	48.18 ± 1.36	45.4 ± 0.88	80.92 ± 2.26^*^	55.57 ± 1.65^#^
TGF-β (ng/ml)	85.92 ± 2.56	85.32 ± 1.29	144.95 ± 2.97^*^	114.60 ± 3.12^#^

The data are shown as mean ± SE; the symbol * indicates a significant difference from the control group, while the symbol # indicates a significant difference from the hypothyroidic group at p < 0.05

#### Lipid profile and hepatorenal function

Table 7 reports the results of serum hepatorenal function and lipogram (ALAT, ASAT, GGT, urea, creatinine, cholesterol, and triglycerides) as well as Calcium level of the study groups. The hypothyroidic group showed a significant elevation in the mentioned physiological measurements in comparison to the corresponding values of the control group. These results concomitants with previous reports^71,72^^73^. Hypothyroidism is known to alter metabolic processes, leading to significant changes in hepatic and renal physiology^74^. The observed elevation in ALAT, ASAT, GGT, urea, and creatinine reflects hepatorenal dysfunction and damage; occasionally elevated GGT monitors impaired bile metabolism. Furthermore, cholesterol and triglyceride obvious elevation may result from altered lipid metabolism due to thyroid hormone deficiency and hepatic dysfunction. Also, increased urea and creatinine levels reflect reduced renal clearance, possibly due to compromised glomerular filtration rate in hypothyroidic conditions. Thyroid hormones influence calcium homeostasis, and hypothyroidism may lead to altered calcium absorption and metabolism, contributing to imbalances in serum calcium levels. In a promising manner, ingestion of hypothyroidic rats with DPEE-HY resulted in valuable improvement in hepatorenal performance, as it markedly restored hepatorenal functions and lipoid profile as well as Calcium level in comparison to the untreated hypothyroidic group. This effect could be attributed to the anti-inflammatory and antioxidant efficiency of DPEE-Hy that reduced oxidative stress and inflammation, protecting hepatic and renal tissues from damage^75^. Similarly, DPEE-HY modulated lipid pathways and normalize cholesterol and triglyceride levels. Also, DPEE-HY might enhance kidney filtration efficiency, leading to lower urea and creatinine levels. The improvement in calcium levels suggests that DPEE-HY may support calcium absorption and metabolism, potentially through endocrine regulation^76^.

**Table 7:** Serum values of hepatorenal functions and lipid profile of healthy, hyperthyroidic, and DPEE-HY treated animals.

	Control	DPEE	Hyperthyroidic	Hypothyroidic ~ DPEE
ALAT (U/L)	132.7 ± 3.37	129.7 ± 2.96	230.7 ± 4.74^*^	148.8 ± 4.92^#^
ASAT (U/L)	174.6 ± 4.91	142.5 ± 2.66	210.9 ± 3.34^*^	155.35 ± 2.81^#^
GGT (U/L)	11.76 ± 0.63	10.95 ± 0.58	15.82 ± 1.09^*^	12.4 ± 0.61^#^
Urea (mg/dl)	38.25 ± 2.41	37.3 ± 2.75	65.82 ± 3.61^*^	50.95 ± 2.57^#^
Creatinine (mg/dl)	0.63 ± 0.059	0.62 ± 0.043	1.35 ± 0.115^*^	0.83 ± 0.031^#^
Chol (mg/dl)	117.3 ± 4.51	114.1 ± 3.31	185.3 ± 4.25^*^	135.9 ± 3.02^#^
Trigl (mg/dl)	96.25 ± 3.29	94.36 ± 1.55	158.1 ± 2.79^*^	121.77 ± 1.78^#^
Calcium (mg/dl)	10.67 ± 0.49	11.43 ± 0.71	7.53 ± 0.42^*^	9.81 ± 0.065^#^

The data are shown as mean ± SE; the symbol * indicates a significant difference from the control group, while the symbol # indicates a significant difference from the hypothyroidic group at p < 0.05

#### Histopathological of thyroid gland

**Fig. 3:**
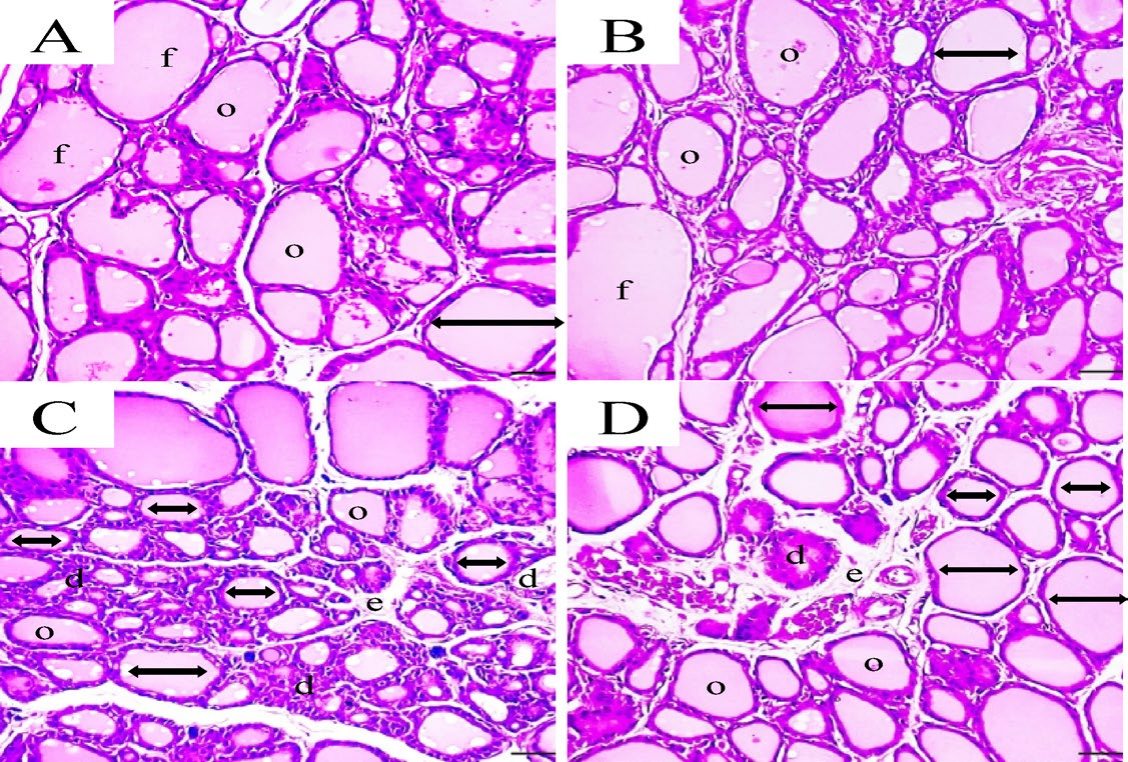
(**A**-**D**). Histopathological examination of the thyroid gland across study groups shows distinct differences. (**A**) The control group exhibited normal thyroid follicles (**f**) with cuboidal epithelial cells, filled with pink colloids (o), and normal blood vessels without interstitial edema. (**B**) The DPEE-fortified yoghurt group displayed mostly normal follicular architecture with slight colloid irregularities, mild blood vessel congestion, and mild interstitial edema. (**C**) The hypothyroidic animals’ group exhibited small, irregular follicles with degraded architecture (**d**), reduced colloid content, numerous apoptotic cells, congested blood vessels, and significant interstitial edema (**e**). Notably, there was a decrease in follicle diameter (double-headed arrow). (**D**) Hypothyroidic animals ingested with DPEE-fortified yoghurt showed improved follicular structure, with more homogeneous colloids, reduced interstitial edema, still congested blood vessels, and fewer apoptotic cells (H&E x400).

The histopathological examination of the thyroid gland of normal control group revealed normal thyroid follicles, characterized by simple cuboidal follicular epithelial cells with eosinophilic cytoplasm and hyperchromatic round nuclei (Fig. 3A). These cells encircled a central lumen filled with pink colloids. Parafollicular C cells appeared as polygonal clusters adjacent to the thyroid follicles. The blood vessels were normal and non-congested, with no evidence of interstitial edema. Similarly, the animals group ingested with DPEE-HY showed that the thyroid follicles largely maintained their architecture, though slight variations were observed. The central lumen was mostly filled with pink colloids, with minor irregularities present. Parafollicular C cells remained as polygonal clusters but were somewhat dispersed. Mild congestion was noted in some blood vessels, and mild interstitial edema resulted in slight separation between thyroid follicles (Fig. 3B). Regarding the hypothyroidism modeled rats, the thyroid follicles displayed small, irregular pleomorphic features and degraded architecture with a desquamated epithelium. Most follicles contained noticeably reduced colloid quantities.

Additionally, congested blood vessels and significant interstitial edema caused pronounced separation between thyroid follicles. However, infiltration of inflammatory like cells could be seen (Fig. 3C); this result confirmed that of Kar et al.^77^. Interestingly, hypothyroidic animals administered with DPEE-HY showed a noticeable improvement in tissue architecture, marked by regular thyroid follicles with intact follicular cells. The colloids appeared mostly homogeneous, with occasional vacuolated areas. Although the blood vessels remained congested, the interstitial edema was milder and reduced compared to the untreated hypothyroidism group. The parafollicular C cells returned to a normal appearance, with fewer apoptotic cells (Fig. 3D). DPEE-HY may be activated TSHR protein expression in PTU-induced hypothyroid animals, possibly in response to the relatively low TSH (H&E x400).

Table 8 presents the histopathological scoring of thyroid tissue sections of the different study groups. Comparing with the control group, the hypothyroidic animals performed a significant increase in follicular degeneration (FD), decrease in colloidal fluid (DCF), atypical follicle epithelium (AFE), and mononuclear cell infiltration (MCI); however, no fibrosis (F) was scored. Interestingly, the administration of hypothyroidic rats with DPEE-fortified yoghurt resulted in a significant reduction in the above deteriorated scores compared to the untreated hypothyroidic group.

**Table 8:** Semi-quantitative scoring for thyroid histopathology of study groups.

Group	FD	DCF	F	AFE	MCI	Total Score
Control	0.2 ± 0.003	0	0	0	0	0.2 ± 0.001
DPEE	0.2 ± 0.002	0	0	0	0	0.2 ± 0.002
Hypothyroidic	2.2 ± 0.006^*^	2.0 ± 0.007^*^	0	1.8 ± 0.005^*^	0.4 ± 0.001^*^	6.4 ± 0.013^*^
Hypothyroidic + DPEE	0.8 ± 0.007^#^	0.6 ± 0.004^#^	0	0.6 ± 0.003^#^	0.2 ± 0.001^#^	2.2 ± 0.004^#^

The scored data are shown as mean ± SE of 5 fields of each group; the symbol * indicates a significant difference from the control group, while the symbol # indicates a significant difference from the hypothyroidic group at *p* < 0.05; FD, follicular degeneration; DCF, decrease in colloidal fluid; F, fibrosis; AFE, atypical follicle epithelium; MCI, mononuclear cell infiltration

## Conclusion

The current study declared that date pollen ethanolic extract-hybrid yoghurt effectively modulated the altered pathophysiological status of thyroid function and architecture; this effect could be a result of the antioxidant efficiency of DPEE constituents that inhibited oxidative stress in the thyroid tissue and probably improved hypothalamic-pituitary-thyroid axis that in its turn preserved thyroid-genesis and maintained normal thyroid function. Consequently, hybridization of yoghurt with DPEE may present a potential modulatory approach for thyroid dysfunction. From a technological standpoint, the inclusion of DPEE hadn’t any negative impact on the yoghurt’s physicochemical properties throughout a 21-day storage period at 5 ± 1 °C.

## Data Availability

All data generated or analyzed during this study are included in this published article.
